# Corrosion-Wear Mechanism of (AlTiV)_100−x_Cr_x_ Lightweight High-Entropy Alloy in the 3.5 wt.% NaCl Solution

**DOI:** 10.3390/ma18112670

**Published:** 2025-06-05

**Authors:** Jiakai Huang, Peng Zhang, Junjie Yang, Wei Li, Qiwei Wang, Jie Li

**Affiliations:** Institute of Advanced Wear & Corrosion Resistant and Functional Materials, Jinan University, Guangzhou 510632, China; 18150722476@163.com (J.H.); tzhangpeng@jnu.edu.cn (P.Z.); junjieyang0612@gmail.com (J.Y.); liweijn@aliyun.com (W.L.)

**Keywords:** lightweight high entropy alloys, oxide film, fatigue wear, corrosion wear

## Abstract

(AlTiV)_100−x_Cr_x_ high-entropy alloys (HEAs) is expected to solve the problem of poor corrosion-wear resistance of lightweight alloys. To elucidate its corrosion-wear mechanism, three (AlTiV)_100−x_Cr_x_ alloys were prepared by vacuum arc melting method by repeating the melting five times at 240 A current.and their microstructures, mechanics, corrosion, wear, and corrosion-wear behaviors were investigated. The results indicate that (AlTiV)_100−x_Cr_x_ is a single-phase with BCC structure and the VEC of Cr5, Cr10 and Cr15 were 4.0, 4.1 and 4.2 respectively. Their hardness increase and toughness and corrosion resistance decrease with the increase of Cr content (Cr5:537.5 HV_0.2_/6.7%/1.86 × 10^−8^ A/cm^2^; Cr10:572.3 HV_0.2_/5.6%/2.09 × 10^8^ A/cm^2^; Cr15:617.6 HV_0.2_/3.8%/2.51 × 10^−8^ A/cm^2^). The wear volume and the corrosion-wear volume of AlTiVCr alloys are mainly caused by the abrasive wear. However, the fatigue wear of AlTiVCr alloys could be exacerbated by a decrease in material’s toughness, corrosion resistance, and an increase in solution corrosivity. Therefore, Cr10 presents the optimal wear resistance in the deionized water, while the optimal corrosion-wear resistance in the 3.5 wt.% NaCl solution is presented by Cr5. Compared to TC4, the wear and corrosion-wear resistance were improved by 56.4% and 65.5%, respectively.

## 1. Introduction

Lightweighting is considered as one of the effective means to reduce fuel consumption and greenhouse gas emissions. Therefore, the lightweight alloys such as aluminum (Al) alloys and titanium (Ti) alloys are widely used in the fields of marine equipment, aerospace, automotive, and petrochemical for their high specific strength [1,2]. However, the utilization of light alloys in the wear-prone conditions is still challenging due to their poor wear resistance resulting from the low hardness [3,4,5,6,7].

Based on the concept of high-entropy alloys (HEA), the lightweight HEAs obtained by using low-density alloying elements such as Al, Ti, and V as the main components, supplemented by a small amount of other metallic or non-metallic elements, are currently an effective means of realizing the combination of low-density and high wear-resistance of metallic materials [8,9,10]. For example, Ye Y et al. [11] prepared an AlTiVZr HEAs with the density of 4.35 g/cm^3^ and hardness of 566.8 HV_0.2_ using the vacuum arc melting. Compared to the conventional titanium alloy—Ti6Al4V (TC4), the wear resistance of the AlTiVZr HEAs is improved by 70.8% due to a 63.8% increase in its hardness. Liu K et al. [12] reported a HEAs of AlTiVCrCu prepared by vacuum arc melting with a density of 5.25 g/cm^3^. Its hardness is up to 871 HV and 1.5 times higher than that of TC4. In conclusion, both AlTiVZr and AlTiVCrCu_0.4_ are multiphase structures whose multiphase structure may favor wear performance in corrosion-free environments, but may negatively affect their corrosion wear resistance.

However, a large number of components, such as the automotive cylinders (Al alloys) and pipeline of pump valve on ships (Ti alloys) are simultaneously subjected to the destruction by wear and corrosion, thereby accelerating their failure [13,14]. It is well known that the damaging behavior occurred at the contact interface during the corrosion wear is affected by the combination effect of corrosion and wear behaviors. As a result, there is a large difference between the wear resistance of a material and its corrosion-wear resistance [15,16]. For instance, it was found by Li J et al. [17] that the wear resistance of low alloy steels could be enhanced by the increasing of TiC content [18]. However, the support of the matrix to the heterogeneous phase could be reduced by their galvanic coupling corrosion, thus weakening the hindering effect of the heterogeneous phase on the micro—cutting of abrasives. Therefore, the corrosion-wear resistance of low alloy steels is disproportionate to the TiC content. Presently, the studies on the wear behavior of (AlTiV) Me HEAs are focused on the corrosion-free environment. A systematic study on the corrosion-wear mechanism of (AlTiV) Me is necessary to ensure the safety and reliability of its application in the corrosion-wear condition.

Among the numerous elements, the introduction of Cr has less effect on the density of (AlTiV) Me high entropy alloy for its low density ~7.19 g/cm^3^. For example, Qiu Y et al. [19] showed that AlTiVCr HEAs has a density of only 5.06 g/cm^3^ but a hardness of 498 HV. In addition, as the mixing enthalpies of AlCr, TiCr, and VCr are respectively −10, −7, and −2 kJ/mol, the precipitation of heterogeneous phases in the AlTiVCr matrix is much low, resulting in a low occurrence of the galvanic coupling corrosion [20]. Meanwhile, the oxide film composed of Cr_2_O_3_ is usually denser and harder, which is beneficial to enhance the material’s wear-corrosion resistance [20,21]. AlTiVCr alloys with non-equivalent atomic ratios can not only have lower densities, but also have a certain increase in hardness. Thus, AlTiVCr HEAs are potentially metal materials with a combination of excellent corrosion-wear resistance and low density.

To reveal the corrosion-wear mechanism of AlTiVCr HEAs, three AlTiVCr HEAs with different Cr contents were prepared by the vacuum arc melting in this study at first. Subsequently, the effects of Cr content on the microstructure, mechanics, corrosion, wear and corrosion-wear behavior of AlTiVCr alloys were investigated by means of X-ray diffractometry (XRD), optical microscopy (OM), scanning electron microscopy (SEM), X-ray photodoton spectroscopy (XPS), compression, electrochemical as well as wear tests.

## 2. Experiments

### 2.1. Materials

In the present study, three (AlTiV)_100−x_Cr_x_ HEAs, (AlTiV)_95_Cr_5_, (AlTiV)_90_Cr_10_, and (AlTiV)_85_Cr_15_, were prepared from Al, Ti, V, and Cr with purity > 99.9% using a vacuum electric arc furnace (Physcience Opto-electronics Co., Ltd., Beijing, China,) (protective gas: N_2_). The specific compositions are shown in Table 1. All samples were repeatedly melted five times by the electromagnetic stirring to achieve a homogeneous composition. In addition, (AlTiV)_95_Cr_5_, (AlTiV)_90_Cr_10_, and (AlTiV)_85_Cr_15_ are abbreviated as Cr5, Cr10, and Cr15 for the description. According to the mixing rules [22], the theoretical density of Cr5, Cr10 and Cr15 are 4.29, 4.40 and 4.51 g/cm^3^, respectively. This is similar to the measurements obtained using the Archimedes method [23] in Table 1. In addition, the most widely used Ti alloy—TC4 has been chosen as a reference to clarify the differences between AlTiVCr alloys and conventional lightweight alloys.

### 2.2. Microstructure and Mechanical Behaviors

The phase composition of AlTiVCr alloys was first measured by X-ray diffractometer (Rigaku, Tokyo, Japan, Ultimate IV) after being ground and polished. The measurement parameters are as follows: target material-Cu Kα, current—40 mA, voltage—40 kV, scanning angle—(20–80°), and scanning speed—5°/min. Afterwards, the specimens were etched in the etchant (5% hydrofluoric acid + 3% nitric acid + 92% deionized water) for 40 s and their microstructure morphologies were observed using OM (Leica, DVM6). The hardness measurements were carried out using the Vickers hardness tester (HXD—1000TM) with a load of 200 gf and time of 15 s. To ensure the reliability, the hardness values used in the present study are the average of 10 measurements. Finally, the compression tests were carried out on a multifunctional testing machine (Norwood, MA, USA, Instron-3369) at a strain rate of 1 × 10^−3^·s^−1^ to obtain the tested alloy’s compression engineering stress—strain curve. Three compression experiments were carried out for each alloy to ensure their reliability. In this experiment, the middle part of the button ingot was selected for microstructure characterization, mechanical property testing and corrosion property test analysis.

### 2.3. Corrosion Behavior

The Tafel polarization curves of the specimens were measured on an electrochemical workstation (GAMRY, Warminster, PA, USA, Interface 1010E) with a three-electrode system. The materials used for the working, reference, and auxiliary electrodes are TC4 and AlTiVCr alloys, saturated calomel electrodes, and platinum sheets, respectively. The electrolyte was 3.5 wt.% NaCl solution. Before the measurement, the tested surfaces were ground and polished to obtain a roughness of Ra 2.0. Subsequently, the specimens were immersed in the 3.5 wt.% NaCl solution for 30 min to ensure a steady state of the open—circuit potential. The scanning range of the potentials used for the tests was from −1.0 V to + 0.5 V, and the scanning rate was 0.5 mV/s. Finally, the corrosion current densities of the materials were obtained from the Tafel polarization curves using the extrapolation method.

### 2.4. Wear Test and Worn Scar Characterization

The wear and corrosion-wear tests were carried out on a reciprocating wear tester (Rtec, San Jose, CA, USA, Rtec—MFT—5000) in mode of ball—on—plane under the temperature of 25–30 °C. To prevent the galvanic coupling corrosion between the friction partners, Si_3_N_4_ ceramic balls with a diameter of 5 mm were used as the opposing wear material. The solution media for the wear test and corrosion-wear test were deionized water and 3.5 wt.% NaCl solution, respectively. The experimental parameters were set as follows: normal load of 10 N, frequency of 5 Hz, test duration of 30 min, and stroke of 5 mm. The roughness of the tested surface was Ra 2.0. Each test was repeated three times for the reliability. After the tests, the loose wear debris on the worn surface was removed using the alcohol solution (ultrasonic cleaning for 10 min) and compressed air. The wear volume was then characterized using a 3D surface profiler(Rtec, CA, USA, Rtec—MFT—5000) combined with the Gwyddion software (64bit). Finally, the worn scar surface was observed using SEM (Phenom XL, Thermo Fisher Scientific, Waltham, MA, USA).

## 3. Results

**Figure 1:**
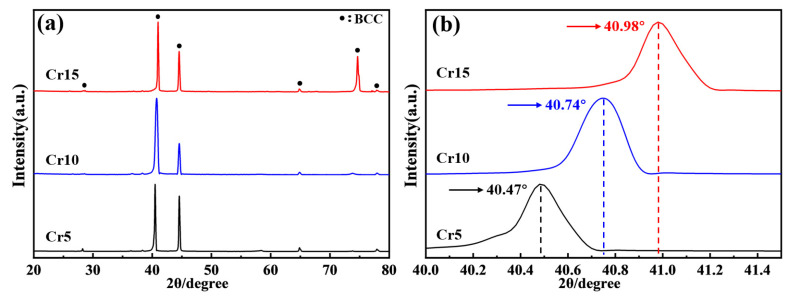
XRD patterns of AlTiVCr alloys: (**a**) the range of 20–80° and (**b**) the range of 40.0–41.5°.

Figure 1 shows the XRD patterns of AlTiVCr alloys. As shown in Figure 1a, only the diffraction peaks of body—center—cubic (BCC) structure could be observed in the XRD patterns of all tested AlTiVCr alloys. A further observation of the diffraction peaks (shown in Figure 1b) indicates that the center position of the most intensive diffraction peak of AlTiVCr alloy gradually shifts to the right with the increase of Cr content. Specifically, it increased from 40.47° for Cr5 to 40.74° for Cr10 and 40.98° for Cr15. It was mainly attributed that the atomic radii of Al, Ti, V and Cr were 0.1434, 0.1445, 0.1311 and 0.1249 nm, respectively. Cr has the smallest atomic radius of the four elements. As a result, the lattice constant of the solid solution decreases due to the increase of Cr content, thereby causing a decrease in the crystal plane spacing. Therefore, based on the Bragg’s equation [24], the diffraction peaks of AlTiVCr are shifted to the high angle with the increasing Cr content.

**Figure 2:**
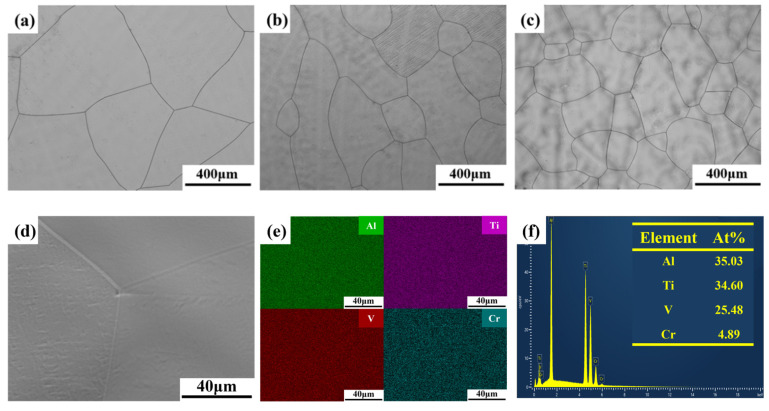
Microstructural characterization of AlTiVCr alloys: (**a**) Cr5, (**b**) Cr10, and (**c**) Cr15 OM image as well as (**d**) SEM images, (**e**) elemental distribution, and (**f**) elemental content of Cr5.

Figure 2 presents the microstructure’s OM and EDS characterization of AlTiVCr alloys. As shown in Figure 2a–c, the grains of all three AlTiVCr alloys are equiaxed without precipitates being observed. Statistics indicates that the grain size of AlTiVCr alloy decreases gradually with increasing Cr content from 486.7 μm for Cr5 to 367.3 μm for Cr10 and 308.4 μm for Cr15. The EDS characterization of Cr5 in Figure 2d,e exhibits a uniform distribution of Al, Ti, V, and Cr elements, with no compositional segregation. The composition of Cr5 is also consistent with the designed values in Table 1. In fact, the compositions of Cr10 and Cr15 are also similar to the designed values. It further confirms that there was no significant burning loss of the alloying elements during their preparations.

**Figure 3:**
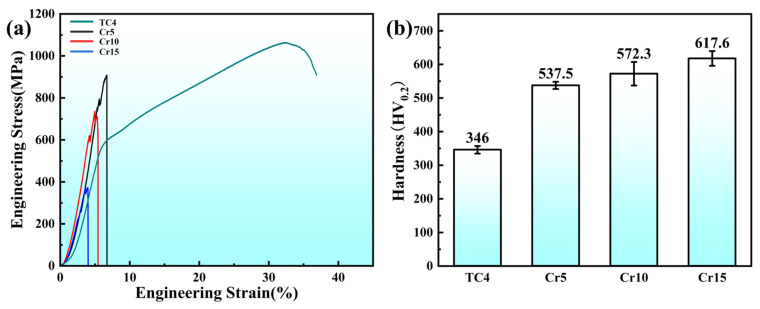

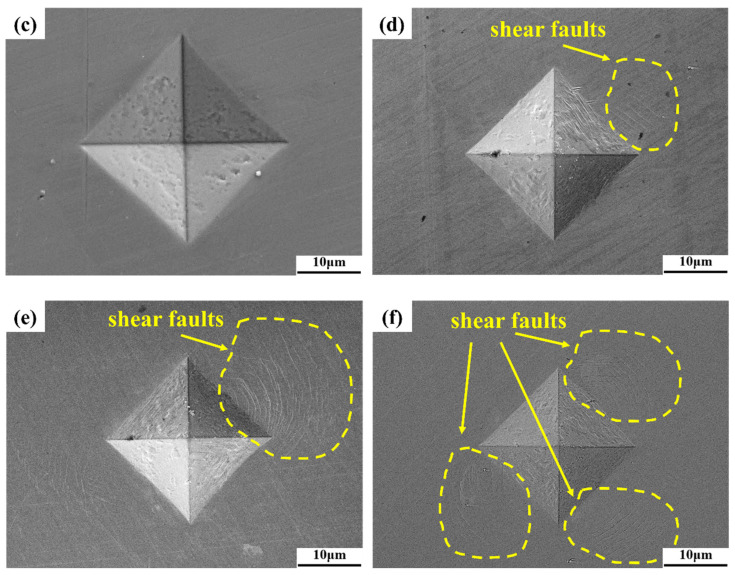
Mechanical behavior of tested materials: (**a**) compressive stress-strain curves, (**b**) Vickers hardness, and indentation SEM images of (**c**) TC4, (**d**) Cr5, (**e**) Cr10, and (**f**) Cr15.

Figure 3 illustrates the characterization of the mechanical behavior of TC4 and AlTiVCr alloys. The compressive stress-strain curves (Figure 3a) and the hardness (Figure 3b) show that the AlTiVCr alloy is much harder than TC4, but with a lower fracture strain. Moreover, the hardness of AlTiVCr alloys gradually increases but its elongation gradually decreases with increasing Cr content. To be specific, the hardness/fracture strain of TC4, Cr5, Cr10 and Cr15 were346 HV_0.2_/36.8%, 537.5 HV_0.2_/6.7%, 572.3 HV_0.2_/5.6%, 617.6 HV_0.2_/3.8%. The indentation morphology in Figure 3c–f suggests that no significant changes are observed in the microstructure around the indentation of TC4. In contrast, a large number of shear faults can be seen around the indentation of AlTiVCr alloy. Moreover, the number of shear faults increases gradually with the increase of Cr content. More shear faults indicate that the material has a poor toughness and a weak resistance to fracture. Therefore, the indentation morphology also indicates that the toughness of AlTiVCr alloy is lower than that of TC4 and decreases gradually with the increase of Cr content.

**Figure 4:**
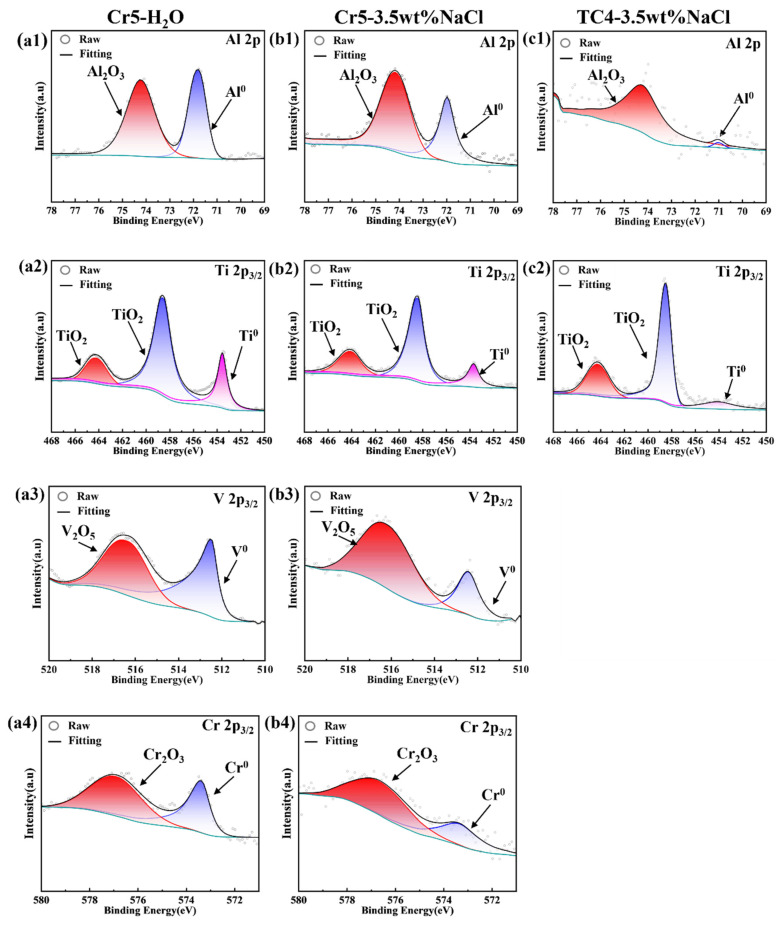
XPS spectra: (**a1**–**a4**) Cr5 immersed in the deionized water, (**b1**–**b4**) Cr5 immersed in the 3.5 wt.% NaCl solution, and (**c1**,**c2**) TC4 immersed in the 3.5 wt.% NaCl solution.

Figure 4 shows the XPS spectra of Cr5 and TC4 after 30 min immersion in the deionized water and 3.5 wt.% NaCl solution. The appearance of the characteristic oxidized peaks indicates that there is an oxidized film on the surface of Cr5 and TC4 alloys. The oxidized film of Cr5 is composed of Al_2_O_3_ + Cr_2_O_3_ + V_2_O_5_ + TiO_2_, while it consists of TiO_2_ + Al_2_O_3_ for TC4 alloys. Furthermore, the oxided film is so thin that X-rays could penetrate it to the metal substrate. Hence, besides the characteristic peaks of the oxides, the characteristic peaks of the metal are also observed in all XPS spectra. However, it is worth noting that in the case of Al 2p, the peak intensity ratios of Al_2_O_3_ to Al in TC4 (immersed in the 3.5 wt.% NaCl solution), Cr5 (immersed in the 3.5 wt.% NaCl solution), and Cr5 (immersed in the deionized water) are 1.42, 1.09, and 0.94, respectively. Considering that the thickness of the oxided film is proportional to the peak intensity ratios of oxide to metal. It can be concluded that the thickness of the oxidized film: TC4 (immersed in the 3.5 wt.% NaCl solution) > Cr5 (immersed in the 3.5 wt.% NaCl solution) > Cr5 (immersed in the deionized water).

**Figure 5:**
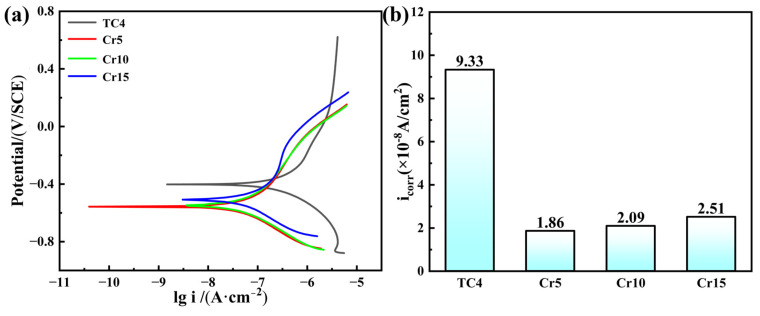
Electrochemical corrosion behavior of AlTiVCr and TC4 in the 3.5 wt.% NaCl solution: (**a**) kinetic potential polarization curves, and (**b**) corrosion current density.

Figure 5 shows the electrochemical corrosion behavior of AlTiVCr and TC4 in the 3.5 wt.% NaCl solution. As shown in the kinetic potential polarization curves of Figure 5a, the corrosion current density of TC4 in the anodic polarization zone was not significantly increased with the increasing potential, which indicates the occurrence of passivation behavior. However, no passivation behaviors were exhibited by the AlTiVC alloys. Then, it could be deduced that the oxidized film on the surface of TC4 is more stable than that of AlTiVCr alloys. However, as shown in Figure 5b, the calculated corrosion current densities of Cr5, Cr10, and Cr15 are 1.86 × 10^−8^, 2.09 × 10^−8^, 2.51 × 10^−8^ A/cm^2^, which are all smaller than that of TC4 (9.33 × 10^−8^ A/cm^2^). The corrosion resistance of AlTiVCr alloys is more excellent than that of TC4, but it decreases with the increase of Cr content.

**Figure 6:**
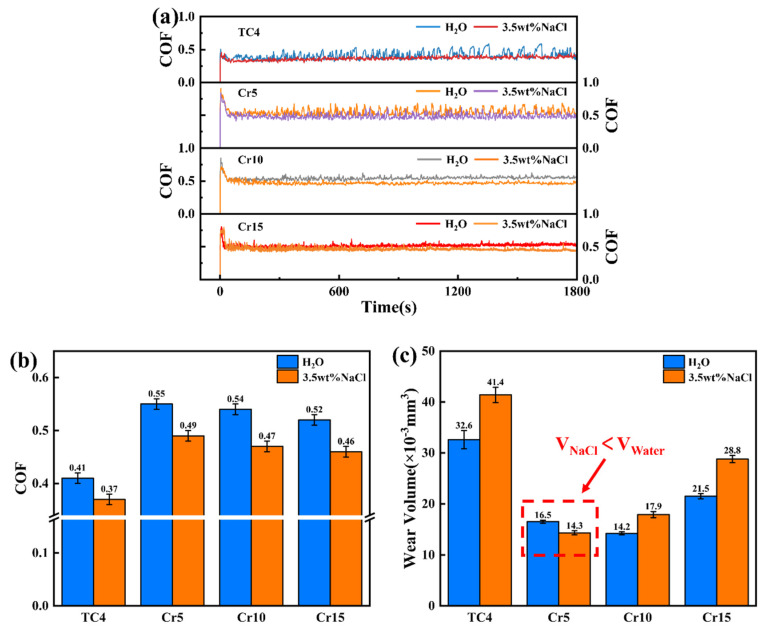
Frictional wear behavior of AlTiVCr and TC4 in the deionized water and 3.5 wt.% NaCl solution: (**a**) the variation of COF with test time, (**b**) averaged COF, and (**c**) wear volume.

Figure 6 shows the frictional wear behavior of AlTiVCr and TC4 in the deionized water and 3.5 wt.% NaCl solution. Figure 6a illustrates the variation of the coefficient of friction (COF) with test time in all conditions. As shown it, the COF all increased sharply at the initial of the test and then decreased slowly to a steady state. It is worth noting that the maximum COF at the initial stage for TC4 is about 0.5, while they are as high as 0.91, 0.82, and 0.76 for Cr5, Cr10, and Cr15, respectively. Figure 6b suggests that the averaged COF of the tested specimens in the 3.5 wt.% NaCl solution are all lower than those in the deionized water. In addition, the COF of AlTiVCr alloys in both solutions decrease gradually with the increase of Cr content but are larger than that of TC4. As for the wear volume, the wear volume of AlTiVCr first decreases and then increases with the increase of Cr content in the deionized water. Cr10 presents the best wear resistance. While it gradually increases with the increasing Cr content in the 3.5 wt.% NaCl solution. Cr5 has the best corrosion-wear resistance. The material loss of AlTiVCr in both solutions was less than that of TC4. In particular, the wear resistance of Cr10 and the corrosion-wear resistance of Cr5 were improved by 56.4% and 65.5% in comparison with TC4, respectively. It is also worth noting that the corrosion wear volume of Cr5 is less than its wear volume, while the opposite is true for the other three materials.

**Figure 7:**
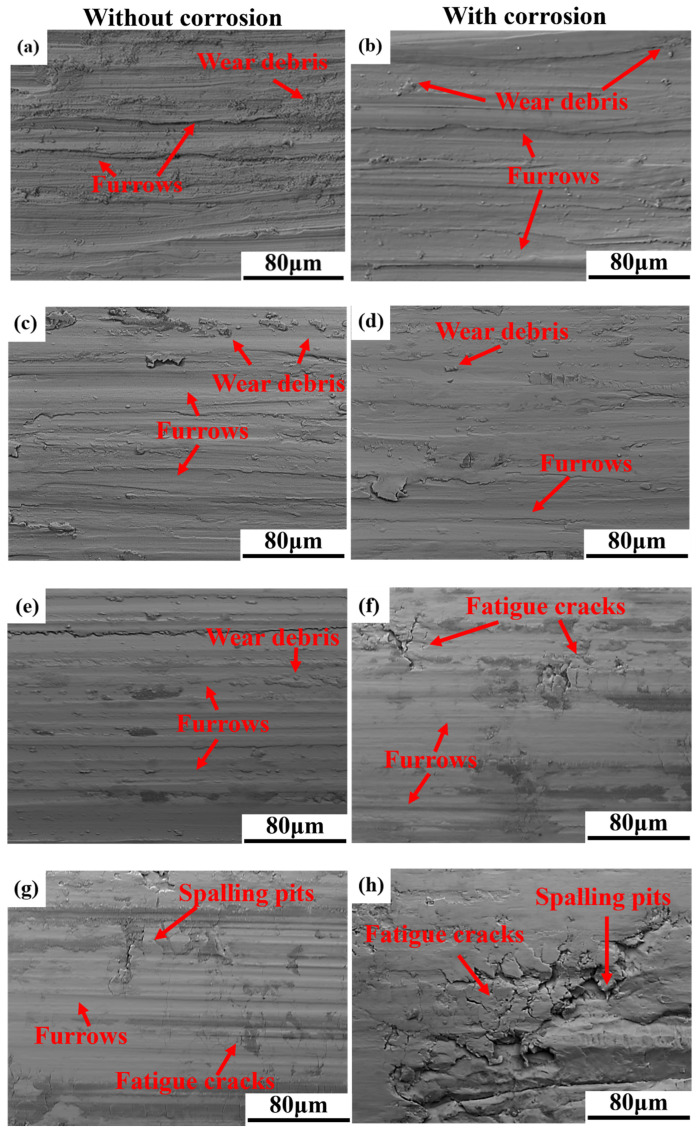
Worn surface SEM morphology of (**a**) TC4, (**c**) Cr5, (**e**) Cr10, (**g**) Cr15 in the deionized water and (**b**) TC4, (**d**) Cr5, (**f**) Cr10, (**h**) Cr15 in the 3.5 wt.% NaCl solution.

Figure 7 illustrates the worn surface SEM morphology of TC4 and AlTiVCr alloys in the deionized water and 3.5 wt.% NaCl solution. From it, a large number of furrows could be observed on the worn surface of all specimens. Therefore, the abrasive wear is the main cause of material loss of TC4 and AlTiVCr alloys in the two solutions. However, it is worth noting that a large number of fatigue cracks could be observed on the worn surface of Cr10 (3.5 wt.% NaCl solution) as well as Cr15 (in both solutions). The intersection of fatigue cracks could lead to material detachment and exacerbate the wear volume. Thus, the wear volume of Cr10 in the 3.5 wt.% NaCl solution and Cr15 in both solutions is affected by the combination of abrasive wear and fatigue wear. It suggests that the fatigue wear of AlTiVCr alloys could be exacerbated by the reduction in its toughness as well as the increase in the solution corrosivity.

## 4. Discussion

AlTiVCr lightweight HEAs are expected to solve the problem of poor corrosion-wear properties of light alloys. In present study, three (AlTiV)_100−x_Cr_x_ HEAs with densities of 4.40 g/cm^3^ were prepared. Then, the microstructure, mechanics, corrosion, wear, and corrosion-wear behaviors of the (AlTiV)_100−x_Cr_x_ alloy were characterized using XRD, XPS, SEM, and electrochemical workstations etc. Then, the influence mechanism of microstructure on the corrosion-wear properties of (AlTiV)_100−x_Cr_x_ alloy would be discussed based on the above characterizations.

### 4.1. Microstructure, Mechanical and Corrosion Properties of (AlTiV)_100−x_Cr_x_

From the XRD pattern (Figure 1) and OM morphology (Figure 2), it is clear that the (AlTiV)_100−x_Cr_x_ alloy consists of a single phase with BCC crystal structure. It has been reported that the crystal structure of the solid solution phase of HEAs is closely related to the valence electron concentration (*VEC*) [25]. For VEC ≤ 6.87 or ≥8, HEAs tends to form BCC and Face-Centered Cubic (FCC) solid solutions, respectively.

(1) $$\begin{array}{c} \mathrm{VEC}=\overset{n}{\underset{i=1}{\sum }}C_{i}{\left(\mathrm{VEC}\right)}_{i}\;\;\;\;\;\;\; \end{array}$$

where VEC_i_ is the valence electron concentration of the ith element of the group. For VEC ≤ 6.87 or ≥8, HEAs is prone to form BCC and FCC solid solutions, respectively. When 6.87 ≤ VEC ≤ 8, the HEA is typically composed of BCC + FCC solid solution. The VEC of Cr5, Cr10 and Cr15 are calculated to be 4.0, 4.1 and 4.2 respectively. Therefore, the crystal structure of (AlTiV)_100−x_Cr_x_ HEAs is BCC.

Regarding the mechanical properties, the lattice distortion of (AlTiV)_100−x_Cr_x_ increases gradually with the increase of Cr content due to the fact that the Cr atomic radius is the smallest among the four elements. Commonly, the lattice distortion could be quantified by the atomic size variability—*δ* [26].

(2) $$\begin{array}{c} \delta =\sqrt{\overset{n}{\underset{i=1}{\sum }}c_{i}{\left(1-\frac{r_{i}}{\sum_{i=1}^{n}c_{i}r_{i}}\right)}^{2}}\;\;\; \end{array}$$

where $c_{i}$ and $r_{i}$ are the atomic percentage and atomic radius of the ith element of the group, respectively. The calculated δ-values of TC4, Cr5, Cr10, and Cr15 are 1.91, 4.81, 5.31, and 5.73, respectively. In addition, the grain size of (AlTiV)_100−x_Cr_x_ alloys decreases progressively with the increase of Cr content. The combination of increased lattice distortion and reduced grain size results in the hardness of (AlTiV)_100−x_Cr_x_ being proportional to the Cr content and much higher than that of TC4. However, the weakening effect of increased lattice distortion on the toughness outweighs the enhancement by grain refinement. As a result, the toughness of (AlTiV)_100−x_Cr_x_ alloy decreases with increasing Cr content.

**Figure 8:**
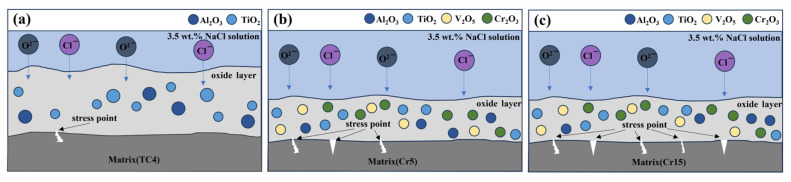
Corrosion mechanism diagram for alloys:(**a**) TC4;(**b**) Cr5 and(**c**) Cr15.

The studies on the effect of V, Al and Cr on the corrosion behavior of Ti alloys have shown that V_2_O_5_, Al_2_O_3_ and Cr_2_O_3_ doping in TiO_2_ oxide film could enhance the densification of the composite oxidized film [27,28,29,30]. Thus, the (AlTiV)_100−x_Cr_x_ alloy has a better corrosion resistance than TC4. However, the excessive internal stresses in the matrix resulted in the failure to form a stable passivation film on the surface of the AlTiVCr alloy. Moreover, the corrosion resistance of AlTiVCr decreases gradually due to the increase of internal stress with the increase of Cr content. The corrosion behavior of AlTiVCr alloys in NaCl solution could be influenced by the combination of stress corrosion and pitting. Thus, it is exacerbated by the increasing internal stress induced by the increase of Cr content and the introduction of chloride ions. The corrosion mechanism diagram is shown by Figure 8.

### 4.2. Wear and Corrosion-Wear Behaviors of AlTiVCr

COF: It increases sharply at the initial stage of the wear test due to the occurrence of adhesive wear for the removal of the oxidized layer on the surface, and then decreases to a steady state due to the formation of oxides. It can be concluded that the oxides on the worn surface have a lubricating function, thereby reducing the COF. From the corrosion behavior, it can be seen that TC4 is more prone to form a thick oxidized layer compared to (AlTiV)_100−x_Cr_x_. Therefore, the maximum COF of TC4 at the initial stage of the wear test is much lower than that of (AlTiV)_100−x_Cr_x_. Furthermore, the COF of (AlTiV)_100−x_Cr_x_ decreases with increasing Cr content as well as the solution corrosivity for their promoting on the oxide’s formation.

Wear and corrosion-wear resistance: From Figure 6, it can be seen that the wear volume and corrosion-wear volume of (AlTiV)_100−x_Cr_x_ vary differently with Cr content. The wear volume decreases and then increases with the increase of Cr content, while the corrosion-wear volume is proportional to it. This can be explained as follows. First, the worn surface image indicates that the material loss of TC4 and (AlTiV)_100−x_Cr_x_ in corrosion-free and corrosive environments could be both is mainly caused by the micro-cutting of Si_3_N_4_ ceramic ball. Second, it is well known that the micro-cutting resistance of a material is proportional to its hardness [31,32,33]. Finally, the fatigue wear of (AlTiV)_100−x_Cr_x_ could be exacerbated by the decreasing toughness of the material as well as the increasing corrosivity of the solution. Therefore, Cr10 presents the best wear resistance while the best corrosion -wear resistance is presented by Cr5.

As the corrosion-wear volume of Cr5 is lower than its wear volume, which may be related to the oxide film. First, the oxides have two effects on the wear volume [34]. Positive aspects: the lubrication of oxides could reduce the wear volume. Negative aspect: the oxides are easily removed by the tangential forces leading to an increase in the wear volume. Second, the formation of oxide film could be promoted by the solution corrosivity. The positive effect of the oxide film on the wear volume outweighs its negative effect, resulting in a lower corrosion wear volume for Cr5 as compared to its wear volume. The opposite is true for TC4, which may be due to the thicker and less dense oxide layer of TC4 compared to Cr5. The corrosion-wear volume of Cr10 and Cr15 is larger than their wear volume, which mainly originates from the worsening fatigue spalling. Moreover, the promotion of corrosive behavior on the crack formation in Cr15 is more pronounced for its poor toughness and corrosion resistance. As a result, the wear volume increment of Cr15 is greater than that of Cr10.

### 4.3. The Link Between Microstructure and Corrosion

**Figure 9:**
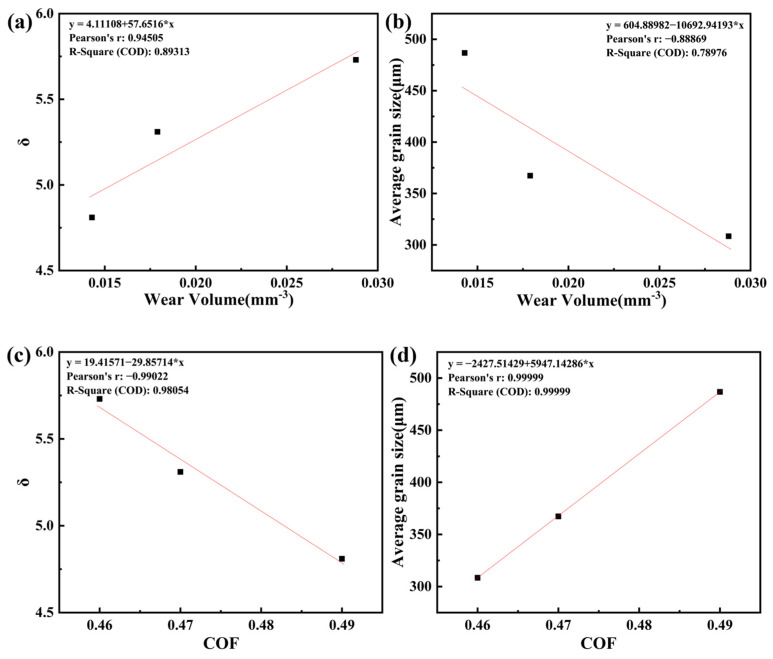
Plot of wear volume, steady state COF versus δ value, and average grain size for AlTiVCr alloys: (**a**) *δ-*wear volume; (**b**) average grain size*-*wear volume; (**c**) *δ-*COF; (**d**) average grain size*-*COF.

Correlation Analysis of Wear and Friction with Alloy Parameters We plotted the given data to examine how wear volume and steady-state COF vary with the atomic size mismatch parameter *δ* and the average grain size. Figure 9a (wear vs. *δ*) shows a clear positive trend: wear volume increases as δ increases from 4.81 to 5.73. Conversely, Figure 9b (wear vs. grain size) indicates wear volume increases as grain size decreases. (larger grains correspond to lower wear in our data). Linear regression was applied to each pair of variables. For wear vs. *δ*, the fit yields R² ≈ 0.89 (*p* = 0.212), and for wear vs. grain size R² ≈ 0.79 (*p* = 0.303). The relatively high R² values suggest a good linear trend, but the *p*-values exceed 0.05 due to the small sample size (n = 3) and thus are not statistically significant at the 95% confidence level. We note that finer grains often confer higher hardness and better wear resistance. The observed increase in wear for the smallest grain sample (Cr15) is therefore somewhat counter-intuitive and may reflect the combined effect of grain size and *δ* in this alloy system. In high-entropy alloys, higher values of *δ* indicate greater lattice distortion: this strengthens the material but may also affect the tribological behavior; our data show that in this case, higher values of *δ* (smaller grains) are associated with more wear, due to the fact that as the value of δ increases, the material hardness, although increasing, increases in brittleness, exacerbating fatigue spalling of the material. Figure 9c plots the steady-state friction coefficient (COF) against atomic-size mismatch (*δ*), and Figure 9d plots the COF against grain size. The COF decreases slightly with increasing δ (negative slope) and increases with increasing grain size (positive slope). The regression results show that R^2^ ≈ 0.98 (*p* = 0.089) for COF versus *δ* and R^2^ ≈ 0.9999 (*p* = 0.0021) for COF versus grain size. The trend in grain size is statistically significant (*p* < 0.05), whereas the trend in *δ* is not (*p* ≈ 0.09) when only three data points are considered. These results suggest that fine-grained, high-*δ* alloys (Cr15) tend to have lower steady-state friction coefficients, while coarse-grained, low-δ alloys (Cr5) tend to have higher steady-state friction coefficients. This is consistent with the general expectation that grain refinement increases hardness and reduces friction, and with reports in the literature of higher coefficients of friction for coarse-grained (CG) samples than for fine-grained (FG) samples [35]. In our data, the fine-grained Cr15 alloy does indeed exhibit the lowest COF (0.46).

## 5. Conclusions

AlTiVCr HEA is a material that potentially combines the low density with an excellent corrosion-wear resistance. To elucidate its corrosion-wear mechanism, the mechanics, corrosion, wear, and corrosion-wear behaviors of TC4, Cr5, Cr10, and Cr15 were investigated. The conclusions reached are as follows:

(1) The AlTiVCr alloy consists of single phase with BCC crystal structure. The increasing lattice distortion and grain refinement led to a gradual growth in the hardness of AlTiVCr (Cr5/537.5 HV_0.2_, Cr10/572.3 HV_0.2_ and Cr15/617.6 HV_0.2_), which are both greater higher than of TC4 (346 HV_0.2_).
(2) The corrosion resistance of AlTiVCr alloy is superior to that of TC4 for its dense composite oxide film composed of Al_2_O_3_ + Cr_2_O_3_ + V_2_O_5_ + TiO_2_. However, the corrosion resistance of AlTiVCr alloys decreases gradually with the increasing Cr content for the growth of lattice distortion.
(3) The wear volume in and without corrosion are both caused by the abrasive wear. However, the reduction in toughness and corrosion resistance as well as the aggravation in solution corrosivity could exacerbate the fatigue wear of AlTiVCr alloys.
(4) In the deionized water, the wear volume of AlTiVCr decreases and then increases with increasing Cr content. Cr10 presents the best wear resistance, which is 56.4% higher than that of TC4.
(5) In the 3.5 wt.% NaCl solution, the corrosion wear volume of AlTiVCr increases with the increase of Cr content. Cr5 presents the best corrosion-wear resistance, which is 65.5% higher than that of TC4.

## Figures and Tables

**Table 1:** The composition and measured density of AlTiVCr HEAs.

Alloys	Al (at.%)	Ti (at.%)	V (at.%)	Cr (at.%)	Ρ (g/cm^3^)
Cr5	35.00	35.00	25.00	5.00	4.29 ± 0.02
Cr10	33.33	33.33	23.34	10.00	4.40 ± 0.04
Cr15	31.67	31.67	21.66	15.00	4.52 ± 0.04

## Data Availability

Data will be made available on request.
